# Gender-based disparities on health indices during COVID-19 crisis: a nationwide cross-sectional study in Jordan

**DOI:** 10.1186/s12939-021-01435-0

**Published:** 2021-04-06

**Authors:** Mohammad Abufaraj, Zaid Eyadat, Mohammed Qussay Al-sabbagh, Abdullah Nimer, Immanuel Azaad Moonesar, Lin Yang, Walid Al Khatib, Ra’eda Al-Qutob

**Affiliations:** 1 Division of Urology, Department of Special Surgery, Jordan University Hospital, The University of Jordan, Amman, 11942 Jordan; 2 Department of Urology, the Medical University of Vienna, Vienna, Austria; 3 Center of strategic studies, the University of Jordan, Amman, Jordan; 4 School of Medicine, The University of Jordan, Amman, Jordan; 5 Health Administration & Policy, Mohammed Bin Rashid School of Government, Dubai, United Arab Emirates; 6 Cancer Epidemiology and Prevention Research, Cancer Control Alberta, Alberta Health Services, Calgary, Canada; 7 Department of Community Health Sciences, Cumming School of Medicine, University of Calgary, Calgary, Alberta Canada; 8 Department of Epidemiology, Center for Public Health, Medical University of Vienna, Vienna, Austria; 9 Department of Family and Community Medicine, Faculty of medicine, The University of Jordan, Amman, Jordan

**Keywords:** COVID-19, Gender, Mental health, Psychological stress, Women health

## Abstract

**Background:**

COVID-19 has an inevitable burden on public health, potentially widening the gender gap in healthcare and the economy. We aimed to assess gender-based desparities during COVID-19 in Jordan in terms of health indices, mental well-being and economic burden.

**Methods:**

A nationally representative sample of 1300 participants ≥18 years living in Jordan were selected using stratified random sampling. Data were collected via telephone interviews in this cross-sectional study. Chi-square was used to test age and gender differences according to demographics, economic burden, and health indices (access to healthcare, health insurance, antenatal and reproductive services). A multivariable logistic regression analysis was used to estimate the beta-coefficient (β) and 95% confidence interval (CI) of factors correlated with mental well-being, assessed by patients’ health questionnaire 4 (PHQ-4).

**Results:**

656 (50.5%) men and 644 (49.5%) women completed the interview. Three-fourths of the participants had health insurance during the COVID-19 crisis. There was no significant difference in healthcare coverage or access between women and men (*p* > 0.05). Half of pregnant women were unable to access antenatal care. Gender was a significant predictor of higher PHQ-4 scores (women vs. men: β: 0.88, 95% CI: 0.54–1.22). Among women, age ≥ 60 years and being married were associated with significantly lower PHQ-4 scores. Only 0.38% of the overall participants lost their jobs; however, 8.3% reported a reduced payment. More women (13.89%) were not paid during the crisis as compared with men (6.92%) (*P* = 0.01).

**Conclusions:**

Our results showed no gender differences in healthcare coverage or access during the COVID-19 crisis generally. Women in Jordan are experiencing worse outcomes in terms of mental well-being and economic burden. Policymakers should give priority to women’s mental health and antenatal and reproductive services. Financial security should be addressed in all Jordanian COVID-19 national plans because the crisis appears widening the gender gap in the economy.

## Introduction

With more than 30 million confirmed cases and one million deaths worldwide, Corona Virus Disease 2019 (COVID-19) is undoubtedly the largest public health emergency in the twenty-first century insofar [1, 2]. This pandemic with the imposed mitigation strategies, lockdowns, and self-quarantine has global repercussions, adversely affecting individuals, communities, institutions, and countries on several levels [3–5].

The accelerated number of confirmed cases exhausts the resources and disturbing the regular service at the healthcare level, converting healthcare facilities into “COVID-19 care facilities” [4]. The role of primary healthcare has been affected, and this includes essential services such as reproductive health and chronic disease regular follow-up visits [3]. The fear of contracting the infection and the common belief that healthcare facilities have COVID-19 cases prevented people from seeking medical advice for perceived non-urgent conditions [3, 4]. Moreover, the intermittent lockdowns and the crisis’s economic consequences have added to the damage and disrupted healthcare providers [5].

Data from previous pandemics demonstrated dissimilar health- and social-related outcomes based on gender. During the 2014–2015 Ebola pandemic, women were more susceptible to contracting the virus, probably, owing to the gender-based role in being first-line caregivers within the families [6]. Women were also more likely to lose their income and lag behind their education, widening the gender gap [6, 7]. During Zika outbreak 2016, there was a noticeable lack of national policies regarding accessibility to antenatal and reproductive health services [8]. COVID-19 is no different, as it seems to inflate gender inequities on different social, economic, and healthcare-related aspects [9]. Almeida et al. demonstrated that women were more susceptible to psychological stress in forms of anxiety, depression as well as post-traumatic stress syndrome. Increasing rates of domestic violence were also reported [10].

According to the United Nations Population Fund Association (UNFPA), 69% of the Jordanian women reported gender-based violence during this pandemic. They were more drastically affected by the pandemic’s psychological and economic burden than men [11]. We have recently reported that more than 45% of women in Jordan suffered from early quarantine-related anxiety, which was significantly higher than men [12]. Lessons learned from previous pandemics demonstrated that failure to address gender disparities in dealing with crises was associated with further drastic consequences at individual and community levels. This effect can be, to a certain extent, mitigated by adopting gender-based strategies.

Therefore, and owing to the unique social structure in the Middle East and North Africa (MENA) region [13], we hypothesize that the COVID-19 crisis is correlated with disparate derangements in several health indicators among adult women and men. We aim to assess gender-based desparities during COVID-19 in Jordan in terms of health indices, mental well-being and economic burden.

## Methodology

### Study setting

This study was conducted in The Hashemite Kingdom of Jordan (HKJ), an upper-middle-income country (11) with a population of 10.6 million [14]. The average per capita income was 4330 (USD) in 2019 [15]. Jordan is composed of twelve administrative governorates belonging to one of three regions: North (Ajloun, Irbid, Jerash, and Mafraq), Centre (Amman, Balqa, Madaba, and Zarqa), and South (Aqaba, Karak, Ma’an, and Tafieleh). Approximately two-thirds (62%) of the Jordanian population reside in the Center region [16]. About 7 out of 10 Jordanians have active medical insurance, which is held by four main sectors; Ministry of health (Covers 80% of them), Royal medical (Military) services, univeristy hospitals, and the private sector [17]. All dependent of health insurance covered individual are covered as well. Even those who do not have active health insurance and need medical care, can seek “exemption/waiver” to get healthcare services paid by the insurance department at the royal court [18].

### Study design and sampling

**Fig. 1:**
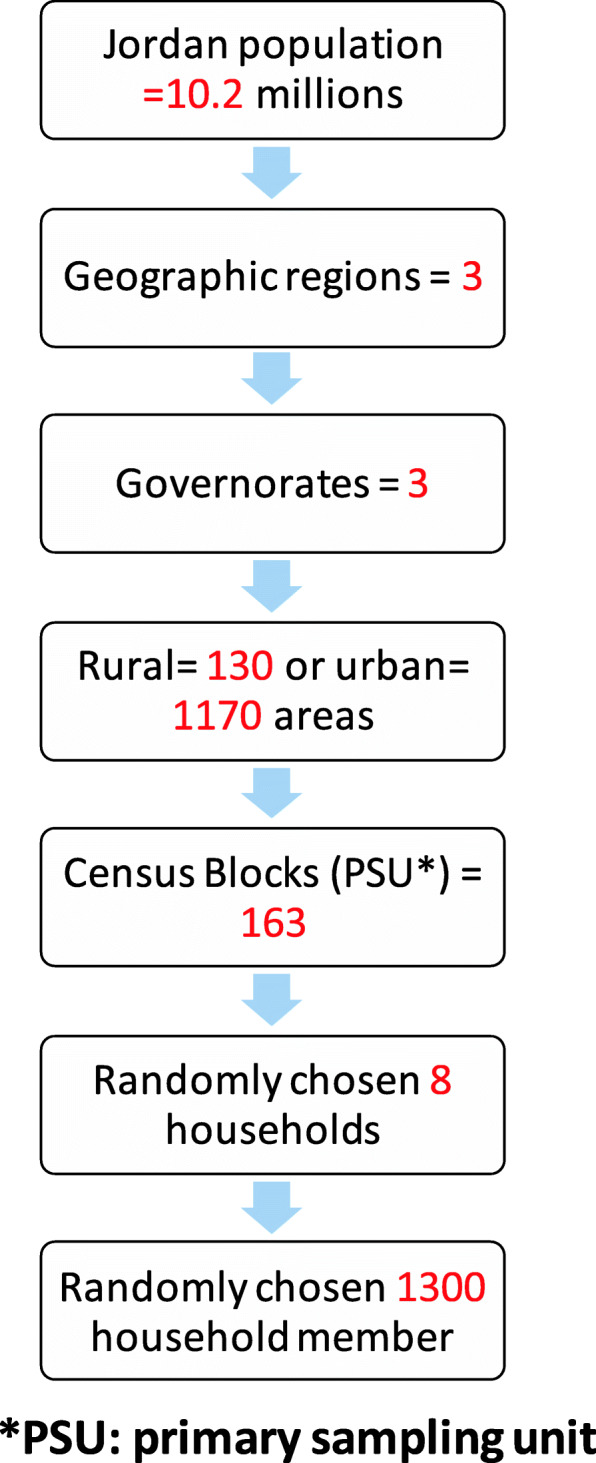
The stratified random sampling protocol to select 1300 participants representing Jordan

This is a cross-sectional survey conducted by the Center for Strategic Studies (CSS), the University of Jordan. The study adopted a multistage stratified cluster sampling design using probability proportional to size to provide valid and reliable representative estimates across Jordan – rural and urban areas, all twelve governorates including the smaller communities, and the geographical distribution including the three regions of the country (Fig. 1). The calculated sample size was 1300 participants. The sample was determined to represent the adult population (≥18 years) living within a household, including people living in Jordan from both genders (women and men) excluding refugees with a margin of error of 5% and a confidence level of 95%.

A household was defined as a group of people living in the same dwelling space who eat meals together, acknowledging the authority of a woman or a man as the head of the household. Places that did not fit the definition of a Jordanian household like student housing, prisons, nursing homes, and factory accommodations were excluded from participation in the study.

### Data collection

Data were collected in May 2020, at that time, HKJ was in a nationwide lockdown with shut down of all educational, economic, and religious activities for almost 2 months [19]. Contact information were obtained through CSS data bases, were they have large phone directory that represent the whole kingdom of Jordan. Following the household selection and obtaining the permission of household residents to participate in the survey, all members who were found eligible were entered into the Census and Survey Processing System (CSPRO) program, which ran a random selection of the household members to participate in the survey. Hence, only one member was able to participate in the study from each household. Out of 1665 individuals contacted initially, 1300 have agreed to participate in the study, yielding a response rate of 78%. The data were collected by trained personnel using a telephone interview, with an average interview time of 16 min. Informed consent was verbally obtained from each participant, and the survey aims were explained at the time of the interview. Data were then entered and coded by interviewers directly into the CSPRO data entry program and then converted o SPSS/Excel format, therefore, there was no missing data. Although the terms “sex” and “gender” are used interchangeably in literature as well as in the Arabic language, the interviewer asked about gender identity in an open-ended, non-judgmental manner while addressing gender roles in the rest of the questions rather than biological sex.

### Measurement instrument

We developed the study instruments with our collaborators at the CSS and Economic and Social Council. The instrument included sociodemographic variables: age (by years), age at first marriage (by years), gender, marital status, pregnancy status, monthly income by Jordanian Dinar (JOD, 1 JOD = 1.41 USD), the region of residence (north, center, or south), number of household members, and the level of education (Illiterate, less than secondary, secondary, and above secondary). It also included health indices: having chronic medical or psychiatric illnesses, having valid health insurance, availability of transport means to hospital, access to hospitals during the quarantine, contraceptive use, access to contraceptive affected during the crisis. In addition, Patient Health Questionnaire 4 (PHQ-4) was used to assess the participants’ mental well-being [20]. The PHQ-4 inquired about two depression and two anxiety symptoms with 4-point Likert scale for each item. It has a total score of 12. The Internal validity for PHQ-4, GAD-2, and PHQ-2 components measured by Cronbach’s alpha are 0.81, 0.74, and 0.65 respectively. The University of California, Los Angeles (UCLA) 3 Item Loneliness Scale was used to assess social support [21]. The UCLA comprises three statements with three possible answers, resulting in a total score of 9; the higher the score, the higher the loneliness and the lower the social support. UCLA-3 has Cronbach’s alpha scores around 0.9, depending on the studied population. To assess COVID-19 stigma, five statements utilizing the Tuberculosis (TB) stigma scale were adopted and modified [22]. These questions were “Some people with COVID-19 fear telling people out of their families that they have the disease”, “Some people think people with COVID-19 are dangerous and disgusting”, “Some people with COVID-19 lose friends when they share the information that they have the disease”, “Some people with COVID-19 lose their jobs when they share the information that they have the disease”, “Some people with suspected COVID-19 symptoms might avoid seeking medical help because other people may see them there”. Each statement had four possible answers on a liker scale; “strongly disagree =0”, “disagree = 1″, “agree = 2″, “strongly agree = 3″ yielding a total score of 15.

### Data analysis

We analyzed the data using STATA (Stata Statistical Software: Release 16. College Station, TX: StataCorp LLC). First, gender differences in demographic variables, healthcare indices, COVID-19 stigma, loneliness, and PHQ-4 score were evaluated using Kruskal-Wallis test. Then, each gender was stratified into three age groups, and differences between each stratum were assessed using Kruskal-Wallis test. Gender differences regarding economic status were analyzed using the chi-square test. Statistically significant results have been defined as *p*-value < 0.05.

A linear regression analysis was used to assess the impact of several sociodemographic variables on the degree of women’s psychological stress. Variables were first evaluated using univariate linear regression analysis. Only significant ones were fitted into the final multivariable-adjusted linear regression model to estimate the beta coefficient (β) and 95% confidence interval (CI).

## Results

### Characteristics of the sample (Table 1)

The distribution of our sample (1300 participants) was proportional to the population size in each governorate and comprised 656 (50.5%) men and 644 (49.5%) women. The mean age of our sample was 43 ± 14.7 years. The 30–50 age group was the most represented, as 281 (42.84%) men and 324 (50.31%) of women were in this group. Most of the participants were married (71.31%), lived in families with 4–6 members (48.62%), and has secondary education or higher (63.8%).

**Table 1:** Socio-economic characteristics of 1300 participants of nationally representative of Jordanian men and women older than 18 years-old according to their age group

Variables		Total (%)	Males (Age group)					Female (Age Group)					Gender differences (***p***-value) ^c^
			Total (%)	< 30	30–50	> 50	***p***-value ^a^	Total (%)	< 30	30–50	> 50	***p***-value ^b^	
	**Total**	1300 (100)	656	166 (25.30)	281 (42.84)	209 (31.86)		644	147 (22.83)	324 (50.31)	173 (26.86)		
**Marital status**													**< 0.01**
	**Married**	927 (71.31)	509 (77.59)	40 (7.9)	262 (51.5)	207 (40.6)	**< 0.01**	540 (83.85)	80 (14.8)	293 (64.3)	167 (30.9)	**< 0.01**	
	**Unmarried**	373 (28.7)	147 (22.41)	126 (85.7)	19 (12.9)	2 (1.4)		104 (16.15)	67 (64.4)	31 (29.8)	6 (5.8)		
**Age at marriage**													**< 0.01**
	**≤18**	142 (13.54)	13 (2.6)	1 (7.7)	5 (38.5)	7 (53.8)	0.6	129 (23.9)	14 (10.85)	51 (39.53)	64 (94.61)	**< 0.01**	
**Region**													**0.04**
	**Capital**	315 (24.23)	147 (22.4)	40 (27.2)	56 (38.1)	51 (34.7)	0.59	168 (26.1)	29 (17.26)	82 (48.81)	57 (33.93)	**< 0.01**	
	**North**	417 (32.08)	228 (34.8)	54 (23.7)	97 (42.5)	77 (33.8)		189 (29.3)	52 (27.5)	88 (46.6)	49 (25.9)		
	**Central**	300 (23.08)	141 (22.4)	37 (26.2)	64 (45.4)	40 (28.4)		159 (24.7)	31 (19.5)	84 (52.83)	44 (27.67)		
	**Southern**	268 (20.62)	140 (21.4)	35 (25)	64 (45.7)	41 (29.3)		128 (19.9)	35 (27.3)	70 (54.7)	23 (18)		
**Income (JOD)**^d^													**< 0.01**
	**≤350**	701 (53.92)	314 (47.87)	81 (25.8)	128 (40.76)	105 (33.44)	0.83	387 (60.1)	75 (19.4)	197 (50.9)	115 (29.7)	**0.03**	
	**350–700**	493 (37.92)	277 (42.23)	64 (23.10)	131 (47.29)	82 (29.60)		216 (33.5)	63 (29.2)	105 (48.6)	48 (22.2)		
	**≥700**	106 (8.15)	65 (9.91)	21 (32.31)	22 (33.85)	22 (33.85)		41 (6.4)	9 (22)	22 (53.6)	10 (24.4)		
**Level of education**													0.63
	**Illiterate**	44 (3.38)	17 (2.6)	1 (5.9)	6 (35.3)	10 (58.8)	**0.01**	27 (4.2)	1 (3.7)	8 (29.6)	18 (66.7)	**< 0.01**	
	**Less than secondary**	425 (32.69)	223 (34)	43 (19.3)	95 (42.6)	85 (38.1)		202 (31.4)	22 (10.9)	97 (48)	83 (41.1)		
	**Secondary**	422 (32.46)	217 (33.1)	63 (29)	107 (49.3)	47 (21.7)		205 (31.8)	56 (27.3)	111 (54.2)	38 (18.5)		
	**Above secondary**	409 (31.46)	199 (30.3)	59 (29.6)	73 (36.7)	67 (33.7)		210 (32.6)	68 (32.4)	108 (51.4)	34 (16.2)		
**Household member**													**0.02**
	**≤3**	280 (21.54)	125 (19.05)	33 (26.4)	37 (29.6)	55 (44)	0.09	155 (24.07)	31 (20)	40 (25.8)	84 (54.2)	**< 0.01**	
	**4–6**	632 (48.62)	319 (48.63)	77 (24.1)	152 (47.7)	90 (2)		313 (48.60)	73 (23.3)	174 (55.6)	66 (21.1)		
	**> 6**	388 (29.85)	212 (32.32)	56 (26.4)	92 (43.4)	64 (30.2)		176 (27.33)	43 (24.4)	110 (62.5)	23 (13.1)		

^a^ Age-based differences among males: Marital status and Education differed significantly according to age in males

^b^ Age-based differences among females: Marital status, Age at marriage, region of residence, income, educational level and number of household members differed according to age in females

^c^ Gender-based differences: Marital status, Age at marriage, region of residence, income, and number of household members differed according to gender

^d^ Monthly household income in JOD = Jordanian Dinar; 1 JOD = 1.41 USD

### Gender differences in healthcare (Table 2 and Fig. 2)

Most men and women participants had health insurance (73%), and the percentages were comparable (73% for men and 72.4% for women). Overall, 60.92% of the sample had an available transport mean to the hospital, with no significant men to women difference (60.82% vs. 61%, respectively). One-fourth of the participants (*n* = 332) had chronic medical or psychiatric illnesses (25.3% in men vs. 25.78% in women, *P* = 1.0); of which, 250 (69%) had access to hospitals during the crisis (men: 68.45% vs. 69.7%, *P* = 0.92). Among married participants, 300 participants (38.4%) used contraceptives, and 252 (32.27%) had access to contraceptives during the COVID-19 crisis, with no significant gender difference (*p* > 0.05). Only 5.95% of married women were pregnant.

**Table 2:** Health indicators of 1300 participants of nationally representative of Jordanian men and women older than 18 years-old according to their age group

Variables		Total (%)	Males (Age group)					Female (Age group)					Gender differences (***p***-value) ^c^
			Total (%)	< 30	30–50	> 50	***p***-value^a^	Total (%)	< 30	30–50	> 50	***p***-value^b^	
**Chronic medical illnesses**	**Yes**	332 (25.54)	166 (25.3)	1 (0.60)	48 (28.92)	117 (70.48)	**< 0.01**	166 (25.78)	1 (0.6)	58 (34.9)	107 (64.5)	**< 0.01**	1.00
**Valid health insurance**	**Yes**	949 (73.00)	483 (73.60)	77 (15.94)	229 (47.41)	177 (36.65)	**< 0.01**	466 (72.4)	98 (21)	228 (49)	140 (30)	**< 0.01**	0.60
**Availability of transport mean**	**Yes**	792 (60.92)	399 (60.82)	110 (27.57)	156 (39.10)	133 (33.33)	**0.05**	393 (61)	94 (23.9)	186 (47.3)	113 (28.8)	0.25	0.98
**Access to hospitals during the crisis**	**Yes**	250 (69)	128 (68.45)	46 (35.94)	28 (21.88)	54 (42.19)	0.9	122 (69.7)	30 (24.6)	50 (41)	42 (34.4)	0.11	0.92
**Contraceptive use**	**Yes**	300 (38.4)	136 (35.42)	14 (10.29)	108 (79.41)	14 (10.29)	**< 0.01**	164 (41.31)	30 (18.3)	134 (81.7)	0 (0)	**< 0.01**	0.81
**Access to contraceptive affected during the crisis**	**Yes**	252 (32.27)	117 (30.47)	14 (11.97)	92 (78.63)	11 (9.40)	**< 0.01**	135 (34.01)	30 (22.2)	102 (77.8)	0 (0)	**< 0.01**	0.29
**Currently pregnant**	**Yes**	20 (5.95)	–	–	–	–	**–**	20 (10)	11 (55.00)	9 (45.00)	0 (0)	**< 0.01**	
**Psychological stress (PHQ-4)**^d^	**0–12**	4.18 ± 3.15	3.7 (3)	4.1 (3.1)	3.9 (3.1)	2.9 (2.8)	**< 0.01**	4.7 (3.2)	4.7 (3)	5.2 (3.2)	3.9 (3.1)	**< 0.01**	**< 0.01**
**loneliness scale**^d^	**0–9**	6.87 ± 2.18	7 (2.1)	6.9 (2.1)	6.9 (2.2)	7.2 (2.1)	0.3	6.8 (2.2)	7.0 (2.11)	6.5 (2.3)	7.1 (2)	**0.01**	0.10
**COVID-19 stigma (S-stigma score)**^d^	**0–15**	7.81 ± 3.08	7.8 (3.1)	7.7 (3.5)	7.8 (3.1)	7.8 (2.9)	0.97	7.9 (3.0)	7.99 (2.9)	7.95 (2.96)	7.6 (3.29)	0.1	0.60

^a^ Age-based differences among males: Chronic medical illnesses, having valid health insurance or transport mean to hospital, contraceptives use and PHQ-4 scores differed according to age in males

^b^ Age-based differences among females: Chronic medical illnesses, having valid health insurance, contraceptives use, pregnancy, PHQ-4 scores and loneliness differed according to age in females

^c^ Gender-based differences: Only PHQ-4 scores differed according to gender

^d^Presented as means (Standard deviations)

**Fig. 2:**
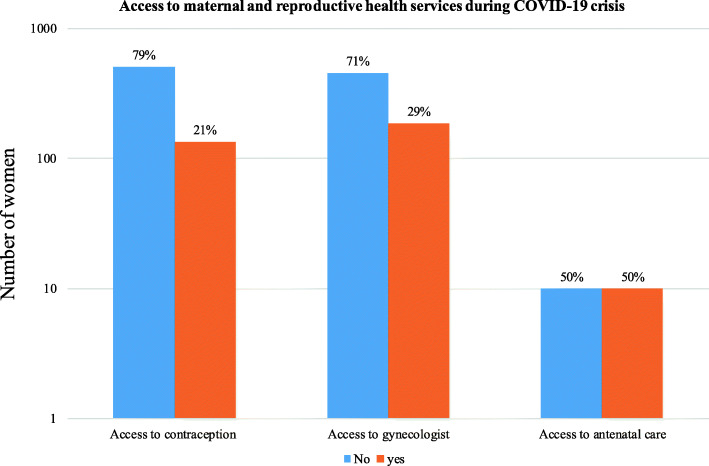
Access to maternial and reproductive health survices in 646 Jordanian women

### Gender differences in economic status (Table 3)

Less than a half of the participants (40.54%) were employed with significantly higher rates in men (63.9%) as compared with women (17.7%) (*P* = 0.01). Only two lost their jobs (0.38%), and one was forced to take leave (0.19%) during the COVID-19 crisis. However, 8.34% of the participants did not get the salary during the COVID-19 turmoil, which was significantly higher in women (13.89%) as compared with men (6.92%) (*p* = 0.02). No significant gender differences were found in other economic variables.

**Table 3:** The differential impact of COVID-19 crisis on the economic status of both men and women

Variable	Total		Males		Females		
	N	%	N	%	N	%	***P*** value
**Had Job before COVID-19**	527	40.54	419	63.9	108	17.7	**0.01**
**Still have Job During COVID-19**							
**Lost job because of COVID-19 crisis**	2	0.38	1	0.24	1	0.93	0.23
**Had been forced to quit my Job because of COVID-19 crisis**	1	0.19	1	0.24	0	0.00	1
**Days in quarantine had been considered as unpaid leaves**	3	0.57	3	0.72	0	0.00	0.76
**Didn’t receive normal salary during COVID-19 crisis**	44	8.34	29	6.92	15	13.89	**0.01**

### Factors affecting mental well-being (Table 4)

Women had a significantly higher mean PHQ-4 score than men (men vs. women: 3.7 vs. 4.7, *P* < 0.01), and there were no significant gender differences in loneliness or COVID-19 related stigma. Table 4 shows a multivariate-adjusted linear regression model of the factors predicting mental well-being. Being older than 50 years was the most robust predictor of lower PHQ-4 scores (vs. < 40, β = − 1.4 95% CI: − 1.8 to − 0.8, *p* < 0.01). Women (β = 0.88 CI: 0.54 to 1.22, p < 0.01), participants who were unmarried (β = 0.7, 95% CI: 0.16 to 1.23, *p* < 0.01), or reported 4–6 household members (vs. < 3, β = 0.77 95% CI: 0.12 to 1.41, *p* = 0.02) were more likely to have higher PHQ-4 scores during the crisis. In a sensitivity analysis (not shown in the table), being unmarried was a significant predictor of having higher PHQ-4 in women (β = 1.2, 95% CI: 0.28 to 1.86, *p* < 0.01), but not in men (β = 1.04, 95%CI: − 0.39 to 1.27, *p* = 0.30). None of the other sociodemographic factors was a significant predictor of the worst mental well-being in the multivariable model.

**Table 4:** Multivariable-adjusted linear regression analysis evaluating the impact of different factors on psycho-social wellbeing of both women and men

Variable	Psychological stress (PHQ-4)		
	β	95% CI^a^	***P*** value
**Gender**			
**Male (ref**^b^**)**			
**Female**	0.88	0.54–1.22	**< 0.01**
**Age (Years)**			
**≤30 (ref)**			
**31–50**	0.53	−0.61 – 0.72	0.88
**≥50**	−1.4	−1.8 - -0.8	**< 0.01**
**Marital status**			
**Married (ref)**			
**Unmarried**	0.7	0.16–1.23	**0.01**
**Region**			
**Capital (ref)**			
**North**	− 0.44	−1.12 – 0.24	0.21
**Central**	0.66	−0.59 – 0.72	0.20
**Southern**	−0.29	−1.02 – 0.43	0.43
**Income (JOD)**^c^			
**≤350 (ref)**			
**350–700**	−0.36	−0.91 - -0.20	0.21
**≥700**	−1.00	−2.02 - -0.92	0.07
**Level of education**			
**Illiterate (ref)**			
**Less than secondary**	0.45	−0.82 - 1.72	0.49
**Secondary**	0.41	−0.89 – 1.70	0.54
**Above secondary**	0.38	−0.94 – 1.71	0.57
**Household members**			
**≤3 (ref)**			
**4–6**	0.77	0.12–1.41	**0.02**
**> 6**	0.61	−0.11 – 1.34	0.10

^a^*CI* Confidence interval

^b^ref.: reference group

^c^ Monthly household income in JOD = Jordanian Dinar; 1 JOD = 1.41 USD

## Discussion

In this representative sample of the Jordanian adult population, approximately two-thirds of the participants had access to health care during the COVID-19 crisis. There was no significant difference in healthcare indices between women and men, but women had significantly higher psychological stress scores. Among women, being ≥50 years old or married was significantly associated with lower levels of stress. Only a few participants lost their jobs during the crisis; however, 8.3% of the respondents reported a reduced or postponed payment that disproportionally affects more women than men.

### Women’s health during COVID-19 pandemic

Equity in healthcare was one of the essential pillars in the national plan of human rights in Jordan [23]. With more than 90 primary healthcare centers that offer maternal and reproductive health services all over the country [24], Jordan has one of the highest women’s life expectancy rates in the region [25]. According to a study conducted by the UNFPA, 83% of Jordanian women were educated and offered a contraceptive means in these primary healthcare centers [24].

According to our study, the use of contraceptives was relatively low among women in the crisis, and more than two-thirds of women were unable to access a contraceptive method. Unfortunately, about two-thirds of women could not visit their gynecologist, and around 64% could not access family planning services. Pregnant represented only 6% of the participating women in this study, but half were unable to access antenatal care. Several authors highlighted such alarming findings and have anticipated increasing maternal mortality and morbidity, especially in middle and low-income countries [26]. This limitation of accessibility is supported by another study conducted by UNFPA stating that because of COVID-19 crisis, around 47 million women globally may potentially lose access to contraception leading to 7 million cases of unintended pregnancies [27]. Another recent cross-sectional study found that the percentage of Jordanian women who did not have access to antenatal care during COVID-19 crisis went up from 4 to 59.5% [28].

Our results showed that around 73% of women have valid health insurance despite during the quarantine, with no difference between genders. Various models have been proposed to explain healthcare services utilization, and one of these models is the Andersen’s Behavioral Model of Health Services Use [29]. According to this model, the decision to utilize a particular health service depends on several factors that are broadly classified into predisposing, enabling, and need factors. One of the essential enabling elements, especially at crises, is the economic factor [30]. Although the vast majority of the study participants did not lose their jobs, a decrease in the salary was reported in approximately 10%. This rate was significantly higher in women, where 15% lost their pay. This rate was significantly higher in women, where 15% lost their pay. Early reports from the pandemic showed that COVID-19 pandemic have multidimensional impact on economy due to loss of human resources (early deaths and disabilities, sick leaves, and drop in productivity), change in consumers’ spending behaviors, and institutional closures [5, 31]. These economic consequences of COVID-19 are polarized, where women, workers in private sector, tourism, industrial, transport and retail workers will suffer more [32–34]. The role of economic and job stability is not limited to their direct effect on healthcare utilization, as they also affect other factors, such as socioeconomic class, access to healthcare, and even health insurance [35].

During the first few months of the crisis, the Jordanian government tackled the pandemic with strict measures [36]. The nationwide lockdown successfully minimized the disease spread initially but suppressing all economic activities eventually resulted in severe consequences. The adverse economic outcomes on people and the healthcare system have started to emerge, requiring national plans to support affected people financially [12]. Therefore, the lockdown was relaxed to salvage the economy despite the disease’s gradual spread and the pressure on health care services [19]. In a country with limited resources, such a vicious cycle of the exhausted healthcare system and collapsed economy carries drastic consequences on people’s well-being.

This public health emergency requires collaborative efforts, including public and private sectors and international organizations. Policy makers should incorporate economists and public health specialist in formulating lockdown policies, where only geographical or temporal “hotspots” are closed without necessarily having nationwide lockdown, thus prioritizing public health without jeopardizing the economy. The primary healthcare services should be integrated actively in national COVID-19 plans, and telemedicine should be utilized to follow up on chronic illnesses and provide essential maternal and reproductive health services. Moreover, media and non-governmental organizations are encouraged to participate in maternal and reproductive health education, increasing the “need” for this service, according to Anderson’s model.

### The impact of the pandemic on mental health

Global crises and pandemics carry adverse effects on the mental well-being of individuals [37]. The psychological stress might manifest as depression, anxiety, or acute stress disorder in the short term and as post-traumatic stress disorder (PTSD) in the long run [37]. An earlier report from Jordan has shown that almost 40% of Jordanians had suffered from quarantine-related anxiety [12]. Our data have demonstrated that psychological stress levels were significantly higher in women, especially in unmarried or younger than 50 years, matching our previous results [12].

In Middle Eastern cultures and countries like Jordan, the different gender roles within the family may put more pressure on women to take care of the ‘children’s education, household care, and health [13]. Also, working women, especially those leading families, might bear more stress because of the fear of losing their income [38]. Another major contributor to mental well-being is gender-based violence, high during COVID-19 crisis and early quarantine [11]. The national healthcare plans should consider women’s mental well-being by offering hotlines and online counseling services and activating all the necessary legislation to protect the vulnerable and those in need. Moreover, working women should be given priority on the financial aid programs and support.

Our results should be interpreted with caution, owing to several limitations. The cross-sectional nature of the study makes it difficult to conclude a cause-effect relationship. In a rapidly changing pandemic, linear time trends should be ideally utilized to illustrate the changes of healthcare access and coverage over time, which is inapplicable in such studies. Moreover, using telephone interviews might result in reporting bias from social desirability or fear from discussing such sensitive topics, as direct interaction offers more trust and empathy. Finally, our data considered medical illness and psychiatric illnesses as one entity, and did not demonstrate the economic consequences of COVID-19 pandemic on different work sectors. Therefore, future studies should address this information in more details. Nonetheless, one of the major strengths of our study is the sampling technique and the tools; the former has been designed to represent all the Jordanian population including different regional, cultural and social backgrounds, and the latter has been developed using validated tools to reflect the current status quo in Jordan.

## Conclusions and recommendations

Our results confirm that the COVID-19 crisis is associated with adverse consequences on both genders in Jordan. However, women are experiencing worse outcomes in terms of access to mental health and income security. Healthcare plans should prioritize women’s mental health, antenatal, and reproductive services, with telemedicine and online counseling services. Policymakers have to offer more financial security to working women and address gender-based violence by reporting and active management. Future research should focus on understanding the factors related to the utilization of healthcare services in more detail, considering the socioeconomic changes emerging from this pandemic and the epidemiological situation changes.

## Data Availability

The data that support the findings of this study are available from The The Center for Strategic Studies but restrictions apply to the availability of these data, which were used under license for the current study, and so are not publicly available. Data are however available from the authors upon reasonable request and with permission of The Center for Strategic Studies.

## Abbreviations

COVID-19: Corona Virus Disease 2019
UNFPA: United Nations Population Fund Association
MENA: Middle East and North Africa
CSS: Center for Strategic Studies
CSPRO: Census and Survey Processing System
PHQ-4: Patient Health Questionnaire-4
UCLA: The University of California, Los Angeles
β: Beta coefficient
CI: 95% confidence interval
PTSD: Post-traumatic stress disorder
